# Design and implementation of adolescent health Latin dance teaching system under artificial intelligence technology

**DOI:** 10.1371/journal.pone.0293313

**Published:** 2023-11-02

**Authors:** Xutao Liu, Kim Geok Soh, Roxana Dev Omar Dev, Wenling Li, Qing Yi

**Affiliations:** 1 Department of Sports Studies, Faculty of Educational Studies, Universiti Putra Malaysia, Serdang, Malaysia; 2 Faculty of Sports and Exercise Science, Universiti Malaya, Kuala Lumpur, Malaysia; Menoufia University, EGYPT

## Abstract

Since various dance teaching systems have attracted much attention with the development of Artificial Intelligence (AI) technology, this paper improves the recognition performance of Latin dance teaching systems by optimizing the action recognition model. Firstly, the object detection and action recognition technology under the current AI technology is analyzed, and the Two-stage object detection algorithm and One-stage object detection algorithm are evaluated. Secondly, the technologies and functions contained in the adolescent health Latin dance teaching system are described, including image acquisition, feature extraction, object detection, and action recognition. Finally, the action recognition algorithm is optimized based on object detection, and the rationality and feasibility of the proposed algorithm are verified by experiments. The experimental results show that the optimization algorithm can search the optimal feature subset after five iterations on Undefine Classes of 101 (UCF101) dataset, but it needs seven iterations on Human Motion Database 51 (HMDB51) dataset. Meanwhile, when using support vector machine classifier, the optimization algorithm can achieve the highest accuracy of motion recognition. Regressive Function, Multinomial Naive Bayes and Gaussian Naive Bayes Algorithms have lower prediction delay, as low as 0.01s. Therefore, this paper has certain reference significance for the design and implementation of adolescent health Latin dance teaching system.

## 1 Introduction

### 1.1 Research background and motivations

Adolescent health problems have always been one of the focuses of social attention. With the development of modern science and technology, adolescents are facing more and more health problems, such as lack of exercise, poor sitting posture and obesity [1]. Dance, as a whole-body exercise form, can provide comprehensive physical exercise and cultivate adolescents’ coordination, flexibility and cardiopulmonary function. Therefore, it is of great significance to design a healthy Latin dance teaching system for adolescents based on artificial intelligence (AI) technology.

AI is an important branch of computer science, which mainly studies how to make machines simulate human intelligent behavior and thinking process. Its wide application in many fields has given it a famous position [2]. For example, in medical diagnosis, AI can provide fast and accurate diagnosis results through big data analysis and machine learning algorithms. In the field of autonomous driving, AI helps cars realize intelligent perception, decision-making and control, thus achieving a safer and more efficient transportation system. In the current work, there are various types of applications of AI. Firstly, based on the application of pattern recognition and machine learning algorithm, features are extracted and pattern recognition is carried out by analyzing a large number of data to realize intelligent decision-making and recommendation. Secondly, natural language processing and speech recognition technology enable computers to understand and process human language information [3]. Finally, in the field of computer vision, AI can extract useful information from images or videos and realize functions such as object recognition and behavior analysis.

To sum up, the design and implementation of adolescent healthy Latin dance teaching system based on AI technology can make full use of the advantages of AI, provide customized teaching content and personalized guidance, help adolescents learn and enjoy dance better, and improve their health level and quality of life. This paper first introduces the target detection and motion recognition under AI technology, then analyzes and optimizes the adolescent health Latin dance teaching system, and finally verifies the performance advantages of this system through experiments. The innovation of this paper is that the system uses computer vision technology and gesture recognition algorithm to analyze the students’ dance in real time and give timely feedback. In this way, students can immediately know whether their dancing is correct, adjust and improve it in time, and improve their learning efficiency. The contribution of this paper can mainly include the following aspects:

1. A teaching system of adolescent healthy Latin dance based on AI technology is designed and implemented. This paper designs and implements a teaching system of adolescent healthy Latin dance combined with AI technology, which can provide teaching content and feedback adapted to students’ abilities and interests according to their individual needs and real-time performance, thus improving learning effect.
2. It provides the functions of motion data analysis and posture monitoring and correction. In this paper, machine learning and deep learning (DL) algorithms are used to analyze students’ motion data, and the functions of motion recognition and evaluation are realized. Meanwhile, posture monitoring and correction algorithms are introduced to help students adjust and correct their dancing posture in real time, prevent sports injuries and improve their dancing skills.
3. The personalized recommendation system and virtual coaching function are realized. The system in this paper recommends suitable Latin dance courses and training plans for students according to their hobbies and physical characteristics through the personalized recommendation system, which increases the fun and motivation of learning. Meanwhile, the function of virtual coach is introduced to provide an immersive learning experience through motion capture technology and augmented reality technology to help students better understand and master dance movements.
4. It provides innovative ideas and application prospects in the field of adolescent health education. The research results of this paper introduce the application of AI technology to the field of adolescent health education, and show its potential benefits and effects through concrete implementation. This is of great significance to further promote the development of adolescent health education and improve the teaching quality and student participation.

Through the above contributions, the research results of this paper have a positive impact on promoting the innovation and development of adolescent health education, and provide valuable reference and enlightenment for the research and practice in related fields.

### 1.2 Research objectives

The research objective is to design and implement an adolescent health Latin dance teaching system based on AI technology and optimize the action recognition model in it. To increase the effectiveness of instruction and student engagement, AI modules with features like posture detection, action correction, and tailored recommendations is deployed. The action recognition model is optimized to increase its precision and scope.

## 2 Literature review

For the research of dance teaching system, Mikaresti and Mansyur (2022) found that dance education has a positive impact on the physical and mental health of adolescents. The research showed that by participating in dance training, adolescents can improve their physical fitness, including coordination, flexibility and explosiveness. In addition, dance also helps adolescents to cultivate self-confidence, emotional expression ability and teamwork spirit. The results showed that dance education can improve the mental health level of adolescents, reduce anxiety, stress and depression, and promote good emotional regulation and social adaptability [4]. Senecal et al. (2020) conducted a three-month dance training program for a group of adolescents. The results showed that the adolescents who participate in dance training have significantly improved their coordination, flexibility and explosiveness. In addition, they also observed that adolescents who participated in dance training had significantly improved in self-esteem, emotional expression and teamwork [5]. Joshi and Chakrabarty (2021) found that AI technology can be applied to personalized counseling and teaching in youth dance teaching. By analyzing students’ learning behavior and performance, the AI system can provide corresponding teaching content and feedback according to their needs and interests, thus improving the learning effect [6]. As for the research of AI, Haq et al. (2021) used long and short-term memory networks to evaluate the impact of modeling the net budget of rainfall, water use and groundwater [7]. Haq (2022) developed and optimized the deep-seated long and short-term memory model of climate to predict the temperature and rainfall in all Himalayan states [8].

However, although dance education has potential in the healthy development of adolescents, previous studies have also revealed some shortcomings. For example, traditional dance teaching methods often lack personalized guidance, and it is impossible to carry out differentiated teaching according to students’ learning needs and ability level. This will cause some students to encounter difficulties in dance learning and not get proper support and guidance. In view of the above problems, this paper has certain advantages. This paper designs a healthy Latin dance teaching system for adolescents by using AI technology, which can provide personalized guidance and recommendation for different students. The system establishes a personalized model by analyzing students’ learning characteristics, physical conditions and progress, and makes a customized dance guidance plan for students according to the model to meet each student’s learning needs.

## 3 Research model

### 3.1 Object detection and action recognition technology under AI technology

**Fig 1:**
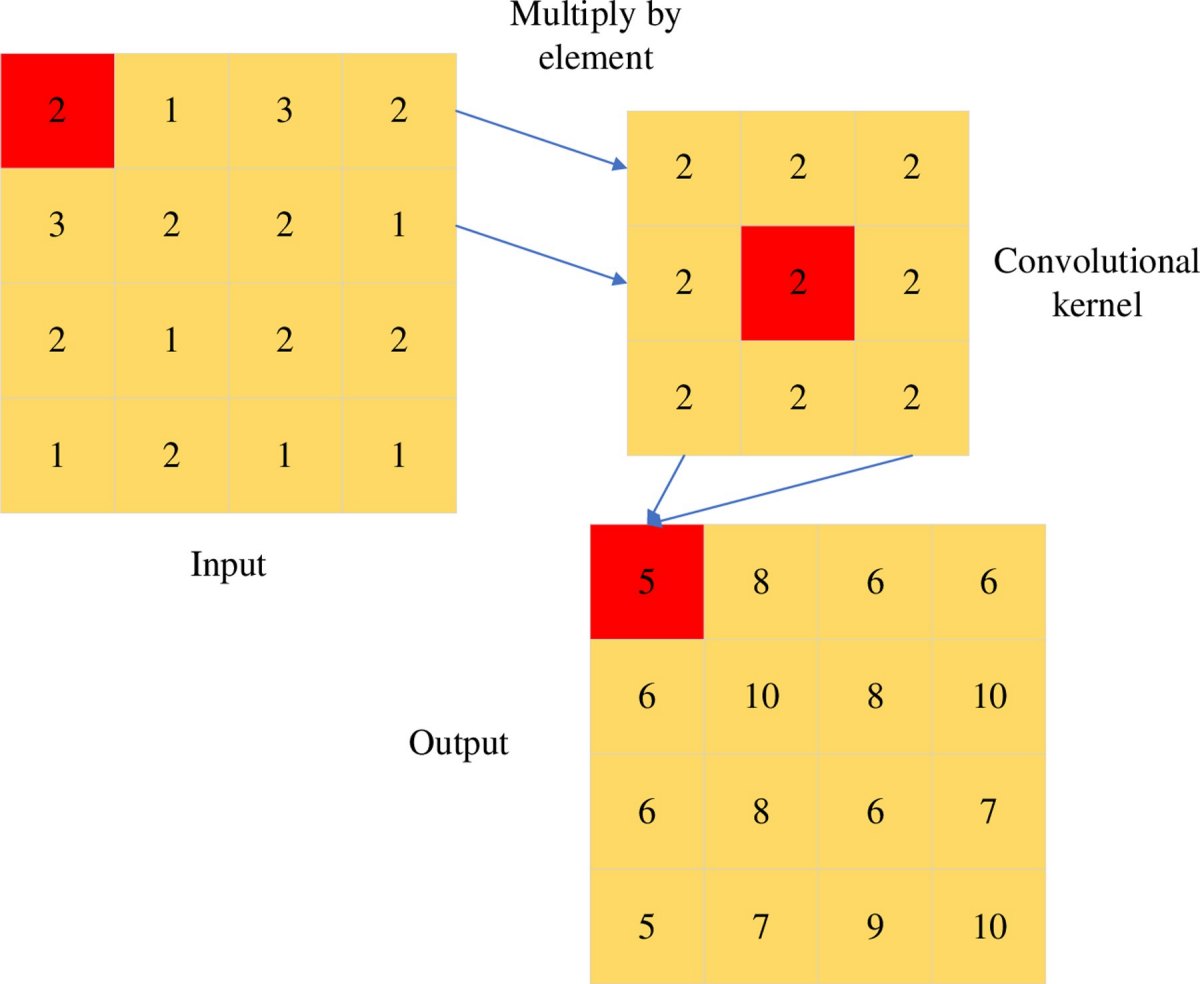
Image convolution calculation process.

The object detection task aims to locate the object of interest in the image and separate it from the image background. It includes two subtasks of object localization and classification, which is the basis of many computer vision tasks. The object detection task has a wide range of applications in image recognition, instance segmentation, pedestrian detection, and unmanned driving [9]. It mainly uses various image processing algorithms to locate and identify specific types of instance objects in digital images and provides basic information for further image understanding. Instance objects in an image can often appear anywhere, with different sizes and shapes. Image processing algorithms are also susceptible to environmental factors, such as perspective relationships, lighting changes, and occlusion, which makes object detection a tricky problem [10]. Traditional object detection algorithms are generally divided into three steps: region of interest selection, feature extraction, and object classification [11]. The region of interest selection is primarily for the purpose of prepositioning foreground objects. Most of the initial research used the sliding window strategy to enumerate the entire image. Its computational efficiency and positioning effect are also closely related to the number of enumerations [12]. Under the development of AI, image convolution computing is currently widely concerned, and its calculation process is shown in Fig 1.

**Fig 2:**
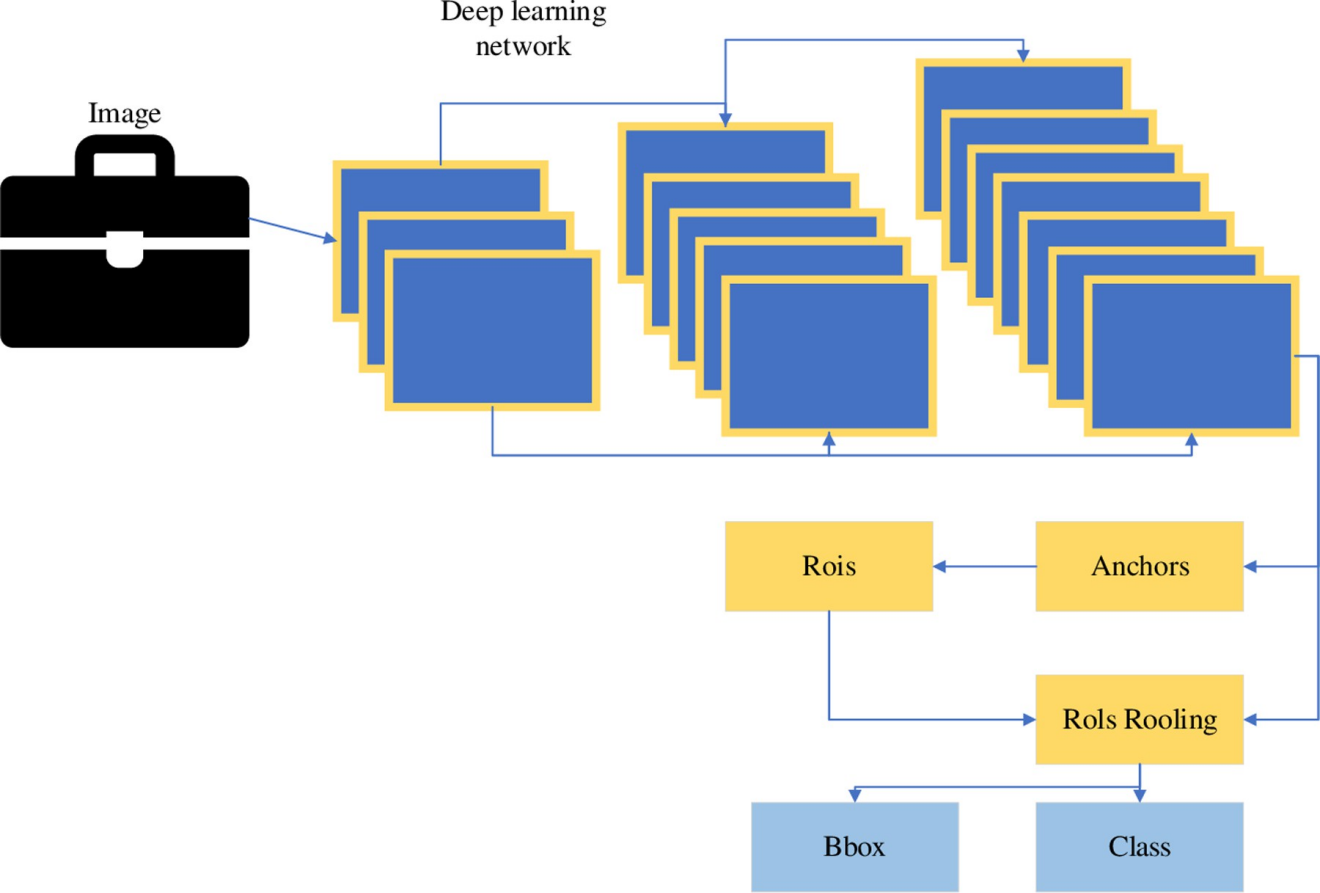
Two-stage object detection algorithm process.

In the era of DL, object detection algorithms can be roughly divided into two types: Two-stage object detection algorithms and One-stage object detection algorithms [13]. The Two-stage object detection algorithm divides the detection process into two steps. First, a category-independent region proposal is generated from the image, which is a sparse set of candidate regions where the target object may exist. Then, features are extracted from candidate regions to determine category labels and optimize target bounding boxes [14]. The specific process is shown in Fig 2.

**Fig 3:**
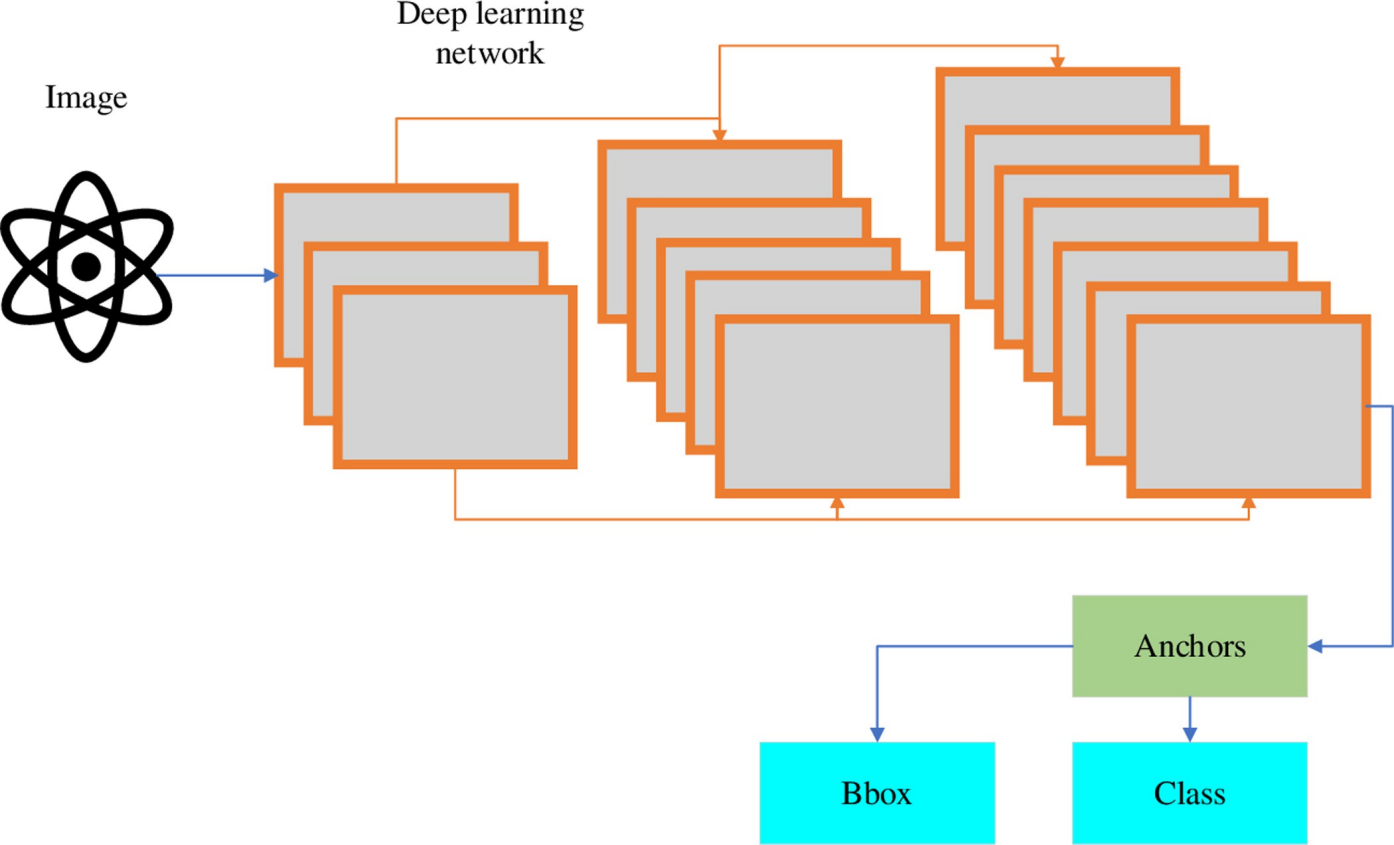
Specific process of one-stage object detection algorithm.

Two-stage object detection algorithm has gradually become the dominant object detection field with its good detection effect. Unlike the Two-stage method, the One-stage object detection algorithm directly regresses the class probability and location coordinates of the instance object without generating candidate regions. The final result can be obtained in a single test [15]. Relatively speaking, the One-stage method has advantages in terms of detection speed. In addition, when targeting is performed, the classifier of the trained classification network is replaced with a regression network. Then, the regression network is trained at various scales to predict the bounding box of the target and fuse the output results at multiple scales [16]. Classification and localization share the feature extraction portion of the network, so classifiers and border regression networks can be run simultaneously at all locations and scales of the image [17]. The specific flow of the One-stage object detection algorithm is presented in Fig 3.

Human Action Recognition (HAR) technology aims to enable computers to understand human action behavior, which is an important research direction in AI. According to the different implementation methods, it can be roughly divided into two types: wearable sensor-based and computer vision-based [18]. The wearable sensor-based method usually requires the subject to wear some relevant sensors (gyroscope and accelerometer) to collect human action data, which is not flexible enough. Also, the application scenarios are relatively limited [19]. In contrast, computer vision-based methods can rely on cameras and other devices to achieve non-inductive data acquisition (images, depth images, and videos). Moreover, one device can collect data from multiple human objects, which has higher flexibility and scalability. It has currently received a lot of study and application. The strategy of relying on video data is the most common among all action detection algorithms based on computer vision [20]. Action recognition using HAR based on DL has become a common practice due to the drive of large data and the superior feature extraction and representation capabilities of deep neural network (DNN) [21]. Deep networks can be used by DL-based techniques to learn behavioral representations of videos from beginning to end. Compared to conventional approaches, it is more adaptable and flexible [22]. The action recognition algorithms based on DL can be broadly categorized as single-flow, dual-flow, and multi-flow networks according to the quantity of input data flows[23].

**Fig 4:**
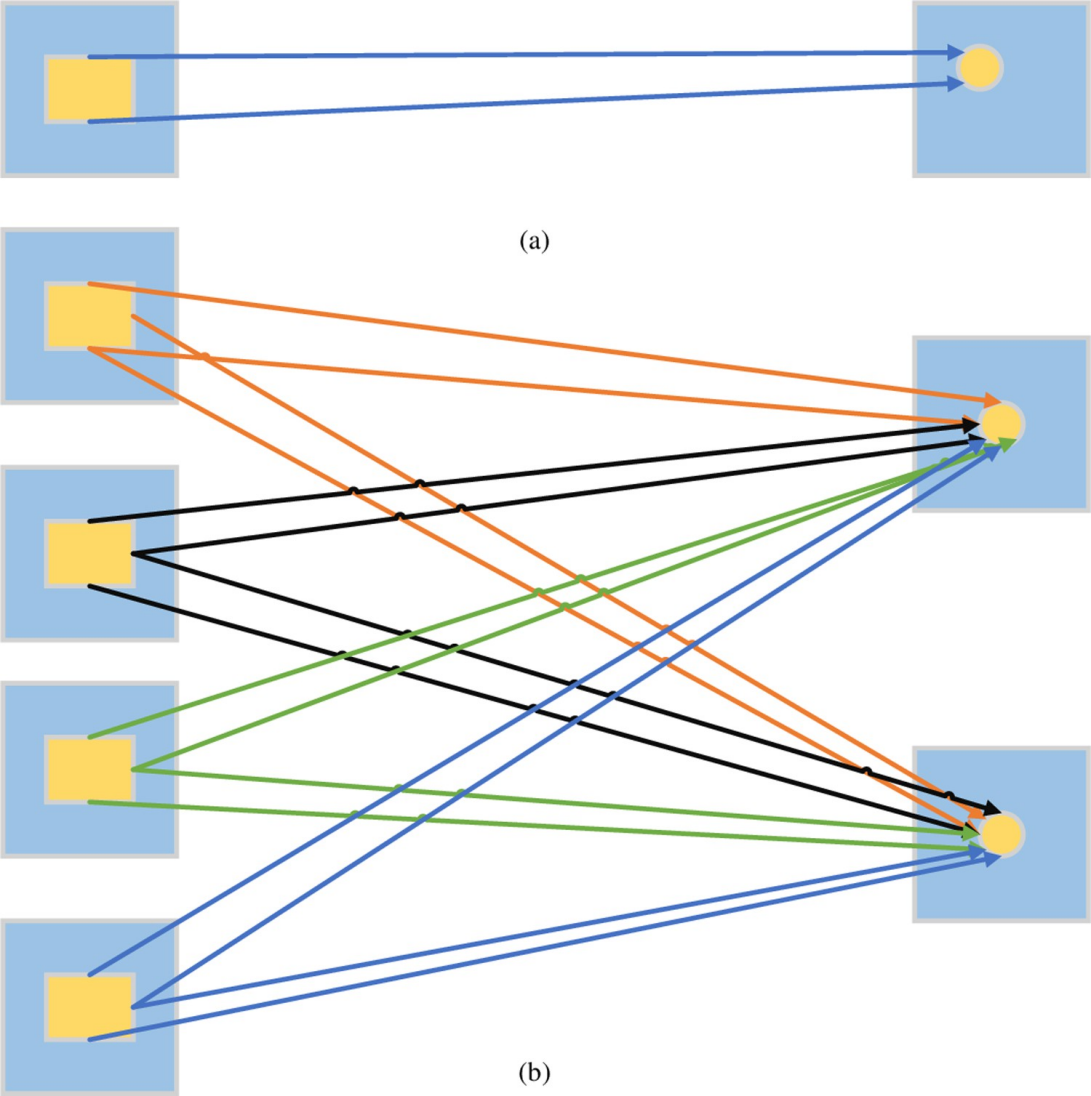
Convolutional operation (a) 2D convolution; (b) 3D convolution.

The 3D convolutional network is a single-flow network widely used in HAR, and its input is a video flow. It is an extension of Two-Dimensional (2D) image convolution with additional temporal dimensions to extract action information from videos [24]. The convolution of the two is given in Fig 4.

**Fig 5:**
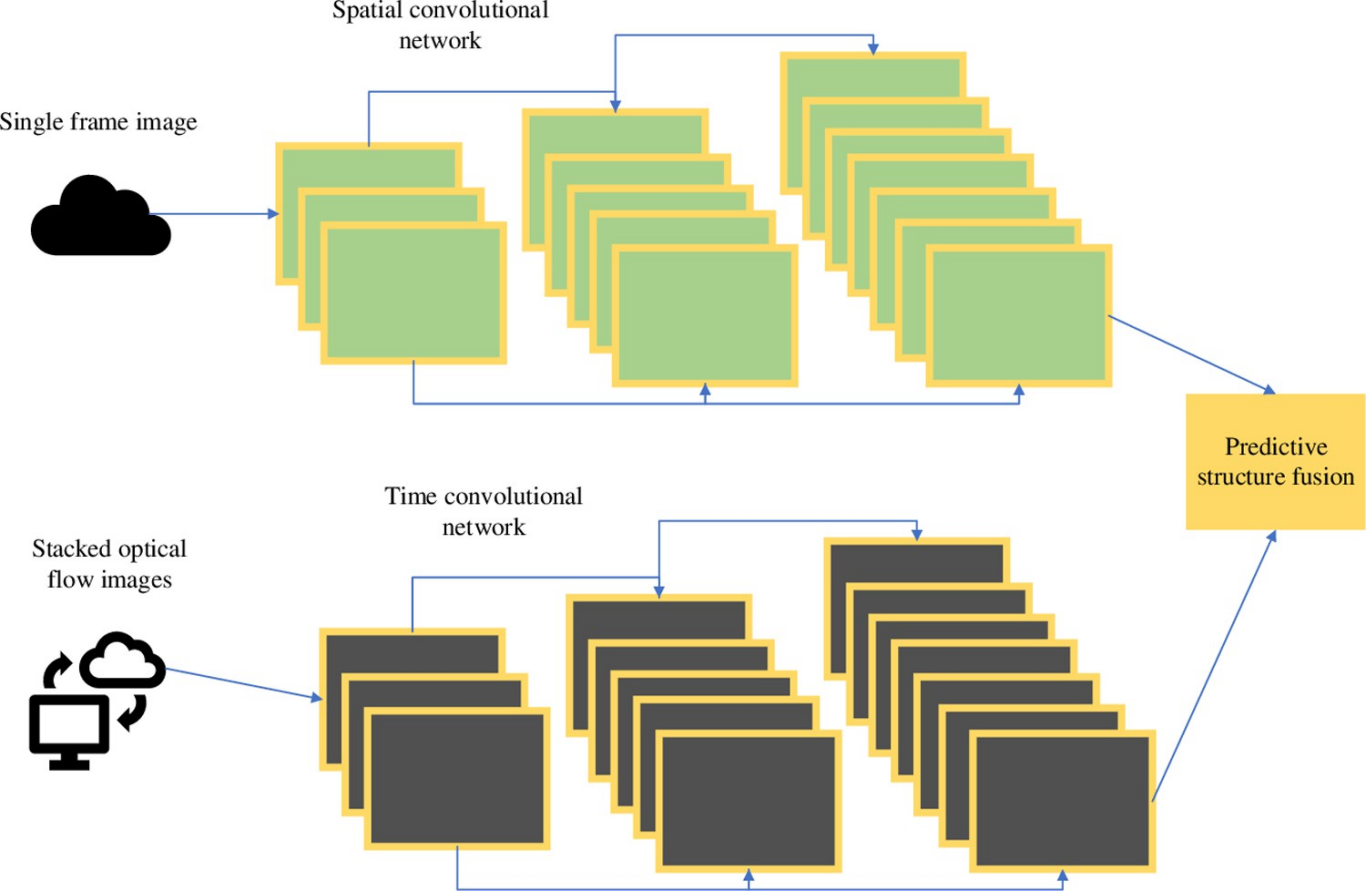
The basic framework of dual flow networks.

Dual-flow networks typically consist of spatial streaming networks and time-streaming networks. The two networks extract features in parallel. Finally, the fusion results of the two networks will be used as the prediction output [25]. The basic framework of a dual-flow network is displayed in Fig 5.

A multi-flow network inputs one more differential image of an action push-stack than a dual-flow network. Finally, the images are processed using a global flow convolutional network [26].

### 3.2 Teaching system and optimization of adolescent healthy Latin dance under AI technology

**Fig 6:**
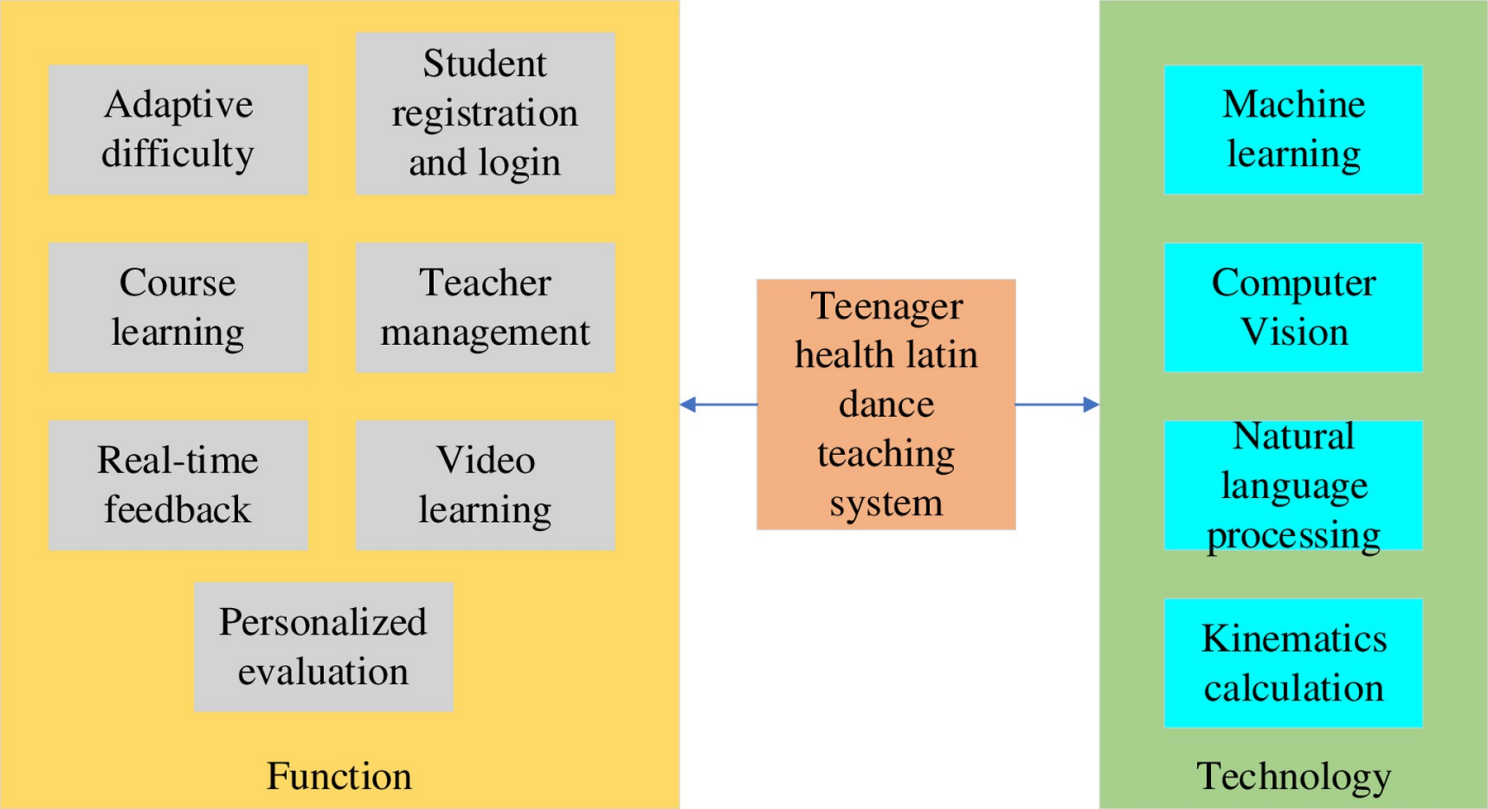
Functions and techniques of adolescent health Latin dance teaching system.

With the rapid development of society and the improvement of people’s living standards, health issues have become more and more important. Physical health and mental health are equally critical for adolescents [27]. However, the fast-paced life of modern society and the popularity of electronic products have led to a lack of exercise among adolescents [28]. Their psychological stress is increased, in addition to how it impacts their physical health. It is now vital to find a solution for the issue of how to better enable adolescents to engage in physical activity and psychological management. An AI-based system for teaching Latin dance to adolescents has developed in this setting [29]. Fig 6 displays the technologies and system features it possesses.

Compared with the traditional Latin dance teaching method, the adolescent health Latin dance teaching system based on AI technology has the following advantages. The first is intelligence. The system can automatically recognize students’ actions and give real-time feedback [30]. It can also automatically adjust the difficulty and pace according to students’ learning progress and ability. The second is personalization. The system can conduct personalized assessments based on students’ performance and data to help students understand their learning and progress. The third is efficiency [31]. The system allows students to master basic dance steps and techniques in a short period to improve learning efficiency [32]. The last is the fun. The system provides rich teaching resources and evaluation systems so that students can feel more interesting and fun in the learning process.

**Fig 7:**
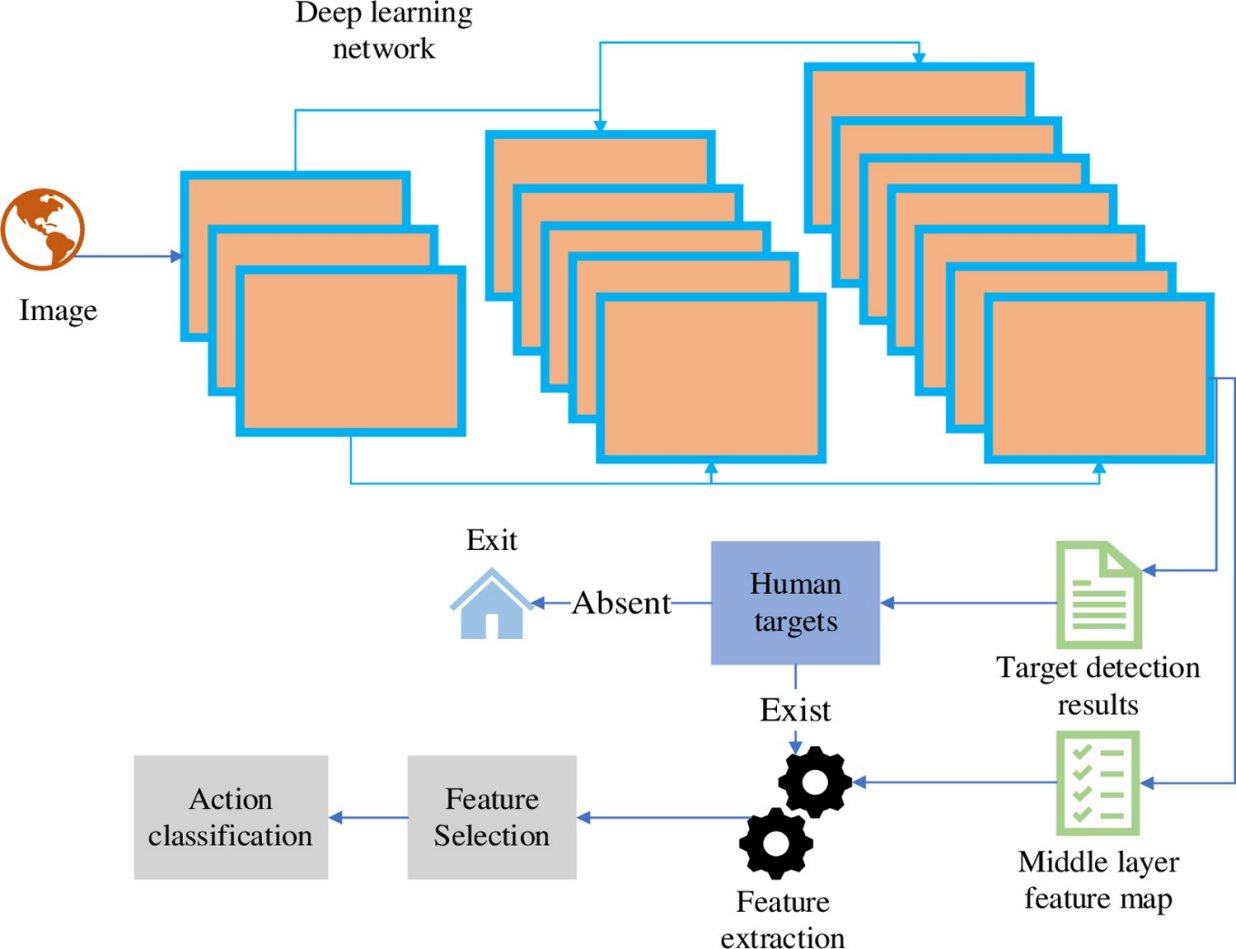
Optimized algorithm framework diagram.

To determine the category and location of the target object, the object detection algorithm based on DL usually uses a deep convolutional neural network (DCNN) to extract some high-dimensional features on the input image that are useful for object detection, such as the local details of the object and the overall environment information of the image [33]. In order to recognize human actions, some abstract high-dimensional features that have been recovered from the object detection model may also be useful. The actions that a person is currently making are always tied to the local specifics of their body, the surroundings, and other items nearby. As a result, the daily HAR method suggested in this research is based on object detection. Fig 7 illustrates the specific algorithm framework.

Aiming at the features obtained by feature extraction and selection, this paper uses machine learning classification method to realize the classification of human actions, and finally builds a complete framework of action recognition algorithm. Firstly, the trained target detection model is used to detect the target of the input image, and the detection results and feature maps of multiple network intermediate layers are obtained. Then, according to the detection results, it is judged whether there are human objects in the image. If there are no human objects, the processing of the input image is ended, and if there are, global features and local features are extracted. Meanwhile, the model is prevented from over-fitting by regularization. The optimized system structure in this paper is shown in Table 1:

**Table 1:** Optimized system structure.

Step	Content
Data collection and processing	Responsible for collecting and processing relevant data, including dance videos, dance steps database, student information, etc. These data will be used to train and optimize the system model.
Posture recognition and analysis	Through computer vision technology and posture recognition algorithm, the dance posture of students is analyzed in real time, including body posture, movement accuracy and so on. The recognition results will be used to provide real-time posture feedback for students.
User modeling and personalized guidance	According to students’ physical conditions, learning characteristics and progress, a personalized model of students is established. Using this model, the system can provide customized dance guidance and recommendation for each student to help them learn and make progress better.
Real-time feedback and guidance	Based on the gesture recognition results and personalized model, the system will provide students with real-time dance analysis and guidance. Through the interface, students’ current action state is displayed, and specific suggestions for improvement are provided to help them correct their mistakes and improve the accuracy of dancing.
Evaluation and tracking of learning progress	Evaluate and track students’ learning progress through AI algorithm. According to students’ performance and progress, the system will provide corresponding learning plans and goal settings to help students effectively manage and plan the learning process.
Social interaction and gamification design	In order to increase the interest and participation in learning, the system can design social interaction function, so that students can learn and make progress together with other students. In addition, gamification elements, such as tasks and reward mechanisms, are introduced to encourage students to continue learning and improve their motivation.
System monitoring and feedback	The system will monitor information such as students’ usage, learning data and user feedback to continuously improve and optimize the design and function of the system. Meanwhile, the system can also provide relevant learning reports and feedback to teachers or parents to intervene and coach students in the learning process in time.

The system can extract features related to actions, including traditional visual features (such as motion trajectory and color histogram) and feature extraction methods based on DL, such as using pre-trained CNN model to transform video frames into advanced spatio-temporal feature representations. In this paper, the algorithm realizes the identification and analysis of adolescents’ healthy Latin dance movements by classifying or clustering the features. In addition, posture analysis and evaluation are be carried out to detect the correctness and quality of movements and provide real-time feedback and suggestions. The deep network model adopts pre-trained CNN, and uses Inception architecture to extract features. In addition, according to the characteristics of the dataset, the experiment chooses the method of moving boundary monitoring for segmentation.

## 4 Experimental design and performance evaluation

### 4.1 Experimental materials

The public datasets selected in this paper are Undefine Classes of 101 (UCF101) dataset and Human Motion Database 51 (HMDB51) dataset, which provide rich and diverse video samples for researchers and provide strong support for developing and evaluating motion recognition algorithms. UCF101 dataset is a large-scale video motion recognition dataset, covering 101 different types of motion categories. The dataset contains 101 action categories, covering all kinds of daily activities (such as making phone calls and listening to music) and sports (such as basketball and football). There are about 13,320 video clips, of which each category contains at least 25 video samples. HMDB51 dataset contains 51 different types of action categories, covering a wide range of daily activities and sports. The HMDB51 dataset contains about 7,000 video clips, of which each category contains at least 100 video samples. In the experiment, the dataset is divided into training set and test set by random division.

### 4.2 Experimental environment and parameters setting

The experimental environment is shown in Table 2.

**Table 2:** Experimental environment.

Device type	Parameter
Processor	Inter(R) Xeon(R) CPU E5-2620 v4 @ 2.10GHz
GPU (Graphics processing unit)	NVIDIA Titan Xp 12GB
Internal storage	128 GB
Programming language	Python 3.6
Technical framework	Py Torch 1.7.0 DL framework
Operating system	Ubuntu 16.04 LTS

K-fold cross-validation method is adopted in the experiment to obtain the classification performance of the motion recognition algorithm on the corresponding datasets. In the experiment, the K value is 10, the data range is selected (0,1), the data dimension is set to 1,000, the learning rate is 0.0001, the training period is 40, the weight attenuation is 0.0001, the size of the input image is 288*192, and the data batch size is 64. In the comparative experiment, this paper chooses five classifiers for experiments, namely Support Vector Machine (SVM), Regressive Function (RF), K-Nearest Neighbor (KNN), Multinomial Naive Bayes (MNB) and Gaussian Naive Bayes (GNB). The penalty term of SVM is 20 and the coefficient of kernel function is 5. The number of RF trees is 20, the maximum depth of trees is 7, and the minimum number of samples required for internal node division is 5. The number of nearest neighbor samples selected by KNN is 10, the weight type is uniform, and the algorithm for calculating nearest neighbor is brute. The smoothing parameter of MNB is set to 0.5, and the GNB parameter need not be adjusted.

### 4.3 Performance evaluation

**Fig 8:**
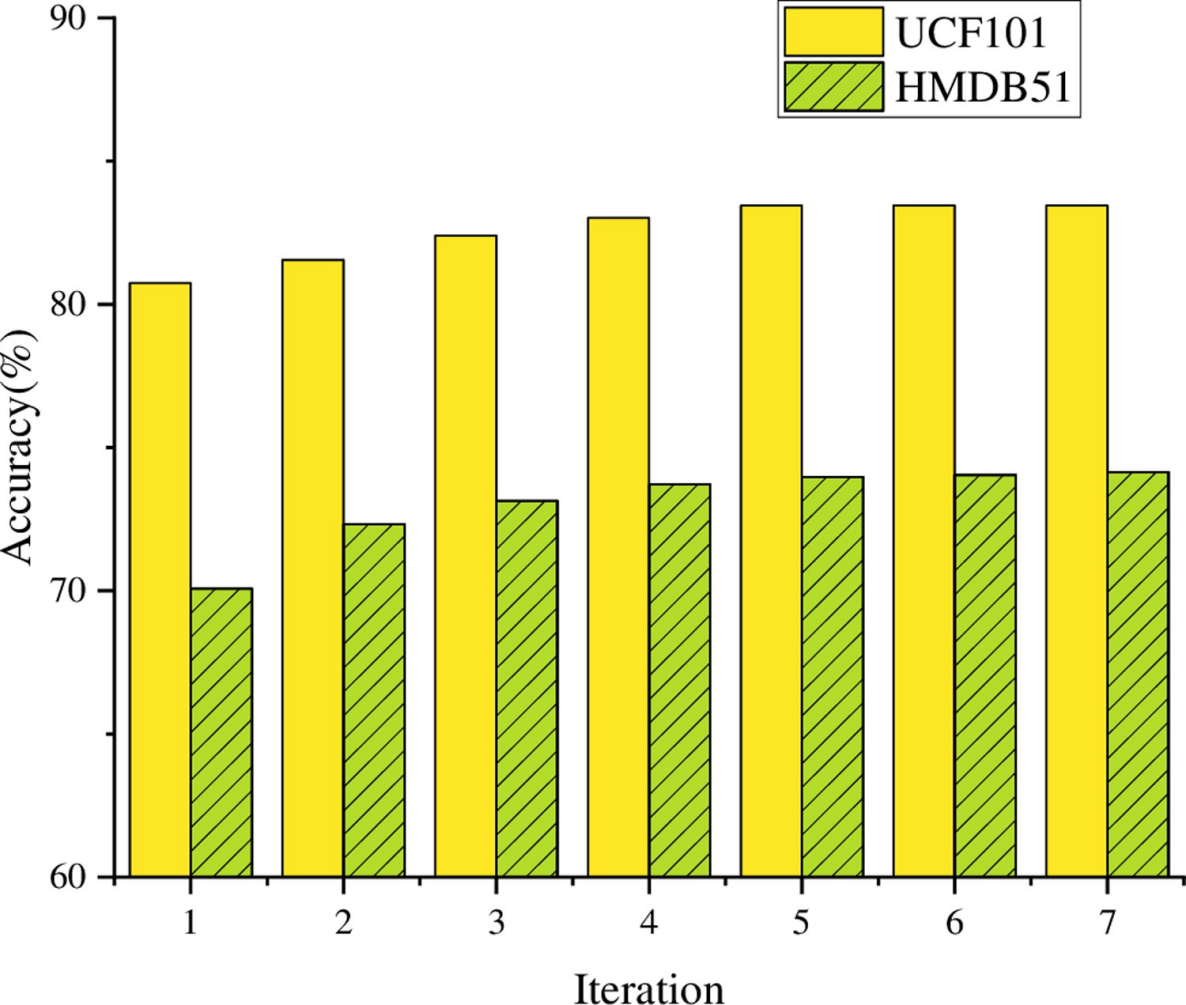
Feature subset search results.

Firstly, the optimization algorithm is used to perform feature selection for 24 features extracted from the object detection model, and the search results of the feature subset are shown in Fig 8.

**Fig 9:**
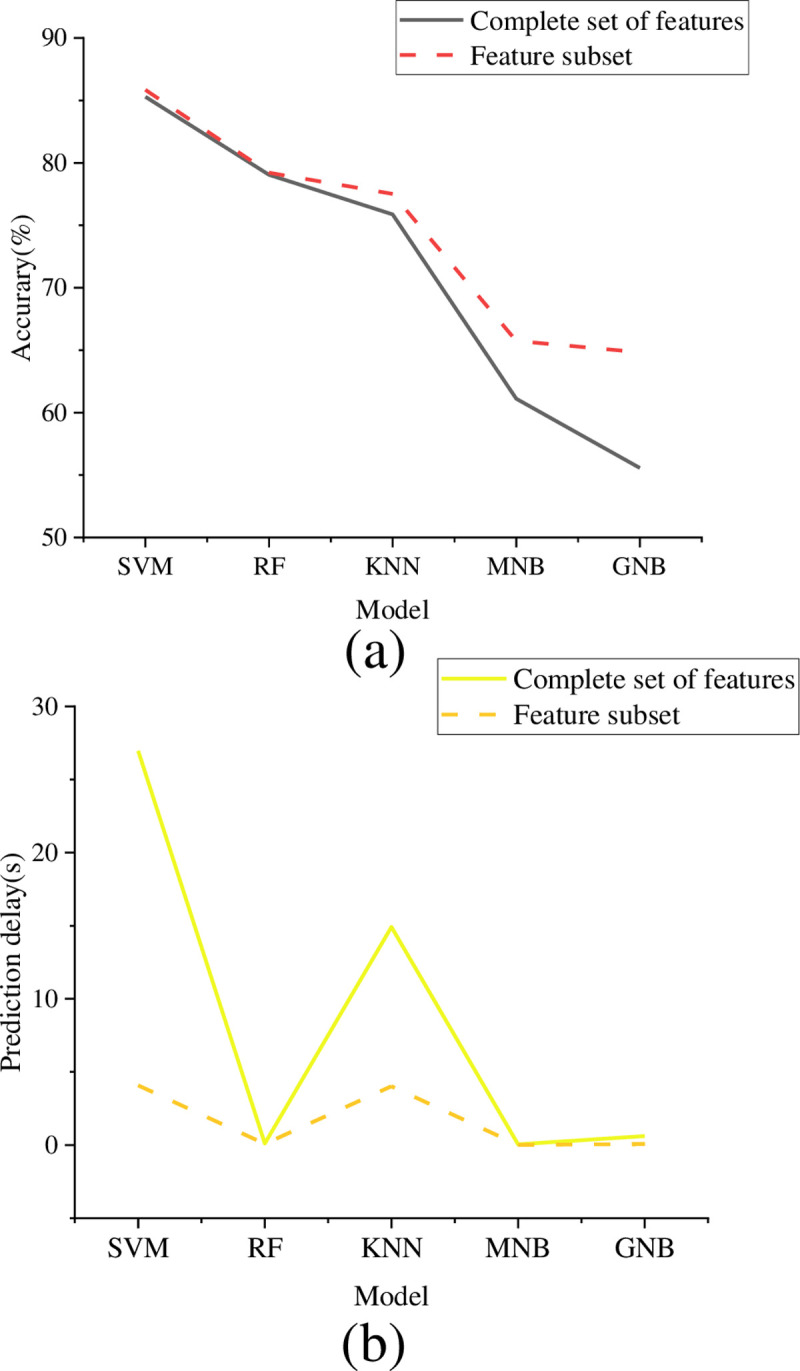
Comparison of action recognition accuracy and prediction delay on the UCF101 dataset (a) Comparison of recognition accuracy; (b) Prediction delay comparison.

It can be observed from Fig 8 that on UCF101 dataset, the optimization algorithm in this paper successfully searches the optimal feature subset after only five iterations, and the corresponding classification accuracy reaches 83.45%. This shows that the algorithm in this paper can find effective feature combinations in relatively few iterations and achieve quite satisfactory classification accuracy. This efficient search process can speed up the training and optimization of the model, which provides a certain practicality for practical application. On the HMDB51 dataset, the optimization algorithm in this paper is sensitive enough, and the optimal feature subset is found after 7 iterations, and the corresponding accuracy reaches 74.14%. Although it is slightly lower than UCF101 dataset, it still has good classification performance. This shows that the optimization algorithm has certain universality on different datasets, and can find a feature combination with strong adaptability to deal with different action behavior recognition tasks. By analyzing the specific information of the searched feature subset, it can be verified that the global features and local features extracted from the target detection model are helpful for the recognition of action behavior. Global features can capture the overall context and spatial relationship of actions, while local features pay more attention to local details and key areas of actions. This combination can provide more abundant and accurate information by using different levels of feature expression, thus improving the performance of action behavior recognition. It should be noted that further analysis of the searched feature subset can deeply understand the attributes and importance of key features, and then improve the algorithm and optimize the model performance. The accuracy and latency of action recognition on the UCF101 dataset are plotted in Fig 9.

**Fig 10:**
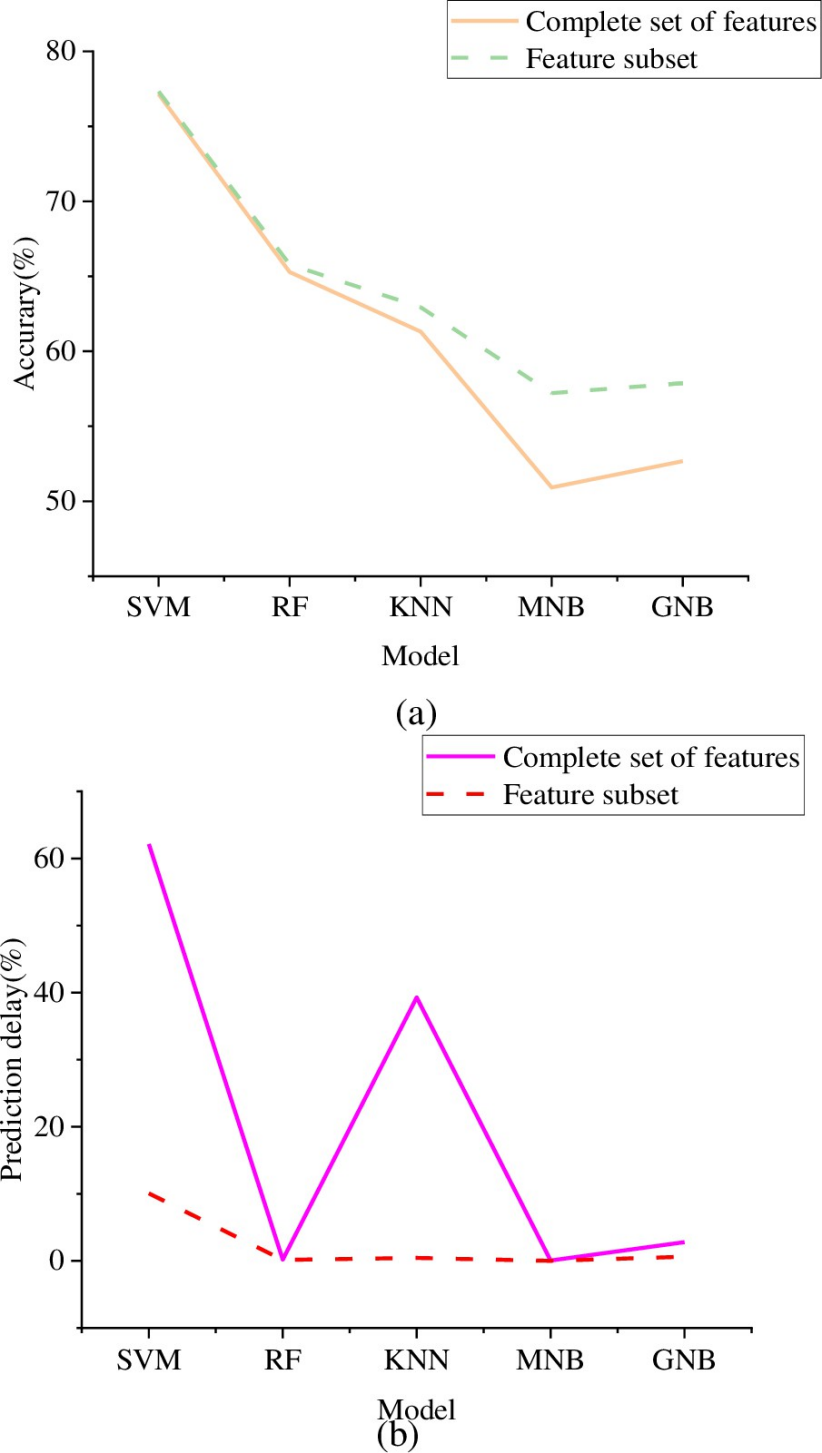
Comparison of action recognition accuracy and prediction delay on the Stanford40 (a) Comparison of recognition accuracy; (b) Prediction delay comparison.

Fig 9 shows that only the middle-layer features extracted from the target detection model can be used on the UCF101 dataset, and a good action recognition effect can be achieved. The highest accuracy of feature set and feature subset can reach 86.31% and 87.85%. This shows that for the action recognition task, the middle-level features already contain enough information to support the classifier to classify accurately. Among the common classifiers, SVM has the highest recognition accuracy, followed by RF, and the recognition accuracy in the feature set is 85.85% and 79.21% respectively. This shows that SVM is the best classifier for the optimization model in this paper, which is suitable for action recognition tasks. RF also has high accuracy, but it is inferior to SVM. In addition, RF, MNB, and GNB have lower prediction delays. This means that these classifiers are faster at processing large-scale data and are more suitable for real-time use cases. Additionally, it can be found that the prediction delay of the model can be reduced through feature selection, for example, the prediction delay of SVM is reduced from 26.96s to 4.08s. Therefore, when selecting a classifier, it is necessary to weigh and choose according to the specific application scenario requirements. The accuracy and latency of action recognition on the HMDB51 dataset are revealed in Fig 10.

The results of Fig 10 show that the experimental results of HMDB51 dataset and UCF101 dataset are compared, and it can be observed that the experimental results of the two datasets are consistent as a whole. On these two datasets, SVM classifier shows the highest accuracy of motion recognition, followed by RF classifier. In addition, RF, MNB and GNB classifiers have lower prediction delay, which means that they have faster response time. However, there are some key differences between the two datasets. Especially on HMDB51 dataset, the accuracy of motion recognition is relatively low. Even for the feature set and feature subset, the highest accuracy rate is only 77.33% and 77.48% respectively. This shows that HMDB51 dataset may be more challenging, in which the action categories are more complex and diverse. Compared with UCF101 dataset, HMDB51 dataset may need more elaborate and complex feature expression methods to capture and distinguish different types of actions.

### 4.4 Discussion

According to the above experimental results, SVM has the highest action recognition accuracy among various classifiers for HAR, followed by RF. RF, MNB, and GNB have lower prediction delays, with latency as low as 0.01s. The feature subset selection process improves the action recognition performance of various classifiers. K-Neighbor and Naive Bayes have a relatively greater improvement. The random forest also has the smallest improvement because the algorithm includes feature selection operations. For SVMs and K-nearest neighbors, the feature subset selection process can improve the classification accuracy while greatly reducing the prediction delay of the classifier. For example, after feature selection, the recognition accuracy of the feature complete set of SVMs will be improved from 86.31% to 87.85%, while the prediction delay can be reduced from 26.96s to 4.08s.

## 5 Conclusion

### 5.1 Research contribution

With the ongoing advancement and use of AI technology, an increasing number of fields have begun to investigate the use of AI technology for innovation and improvement. Adolescent health education is an important field, and healthy Latin dance, as a popular fitness exercise strategy, has been widely used in it. As a result, this paper first examines object detection and action recognition technology under AI technology. Secondly, it describes the technology and functions incorporated in the adolescent health Latin dance instruction system. Finally, the action recognition algorithm is optimized based on object detection, and the suggested algorithm’s effectiveness is validated by testing. The experimental results show that the optimization algorithm can search for the optimal subset of features on the UCF101 dataset after five iterations. On the HMDB51 dataset, the optimization algorithm will search for the optimal subset of features after seven iterations. On different datasets, SVM has the highest action recognition accuracy, followed by RF. RF, MNB, and GNB have lower prediction delays, with latency as low as 0.01s.

### 5.2 Future works and research limitations

This paper studies the recognition of actions in the adolescent health Latin dance teaching system. It locates human body targets in images. Then, action recognition is performed on human targets. The current adolescent health Latin dance teaching system is mainly based on AI technology, but there is still a lot of room for improvement. In the future, more advanced ML algorithms and DL models can be considered to enable the system to more intelligently identify students’ actions and give targeted guidance and suggestions. To enable students to participate more actively in the teaching of healthy Latin dance, the future system needs to add more interactivity and interest.

This paper also has many shortcomings. This paper focuses on specific feature selection and extraction methods. Future research can consider exploring different feature selection and extraction technologies to improve the performance and accuracy of the motion recognition algorithm. It is only a preliminary attempt to select the parameters of five classifiers at the same time, and then the parameters can be further optimized and adjusted to obtain better classification results. In addition, people can try to use other advanced machine learning algorithms or DL models to improve the performance of motion recognition. In the future research, deep neural network can be deeply studied and applied to realize the task of motion recognition. For example, a DL model such as CNN or RNN is used to automatically learn action features from video sequences. It is also possible to explore the fusion of data from multiple sensors (for example, depth camera, inertial measurement unit, etc.) to improve the performance of motion recognition. Multi-modal fusion can provide rich information, thus improving the accuracy and robustness of classification.

## Supporting information

S1 Data (XLSX)
